# DETERMINANTS OF PHYSICAL AND MENTAL HEALTH AMONG LGBT OLDER ADULT CAREGIVERS

**DOI:** 10.1093/geroni/igz038.1248

**Published:** 2019-11-08

**Authors:** Charles Emlet, Charles Emelt, Hyunzee Jung, Hyun-Jun Kim, David La Fazia, Karen Fredriksen-Goldsen

**Affiliations:** 1 University of Washington, Tacoma, Washington, United States; 2 University of Washington, Seattle, Washington, United States

## Abstract

Caregivers experience chronic stress that can negatively affect their health, resulting in lower quality of life. This study aims to understand the physical and mental health of caregivers by examining modifiable risk and protective factors. Life experiences of LGBT older adults are considered. In the longitudinal study, Aging with Pride: National Health Aging, Sexuality and Gender Study, approximately one third (31%, n=754) of LGBT older adults (50+ years) provide care: 38% to partner/spouse, 29% friends, 20% other relatives, and 8% ex-partner/ex-spouse. Linear regression models examined the effects of risk and protective factors at T0 on health outcomes at T2. Mastery, physical activity, and nutrition predicted better physical functioning, lower mental distress, and higher health-related quality of life (HRQOL). Social participation and support, community engagement, and lower identity stigma predicted better mental health. Microaggression predicted lower physical HRQOL. Interventions enhancing protective factors may promote physical and mental health of LGBT caregivers.

